# Elevated serum RAS p21 is an independent prognostic factor in metastatic breast cancer

**DOI:** 10.1186/s12885-018-4282-0

**Published:** 2018-05-08

**Authors:** Malgorzata Banys-Paluchowski, Tanja Fehm, Wolfgang Janni, Bahriye Aktas, Peter A. Fasching, Sabine Kasimir-Bauer, Karin Milde-Langosch, Klaus Pantel, Brigitte Rack, Sabine Riethdorf, Erich-Franz Solomayer, Isabell Witzel, Volkmar Müller

**Affiliations:** 1 Department of Gynecology and Obstetrics, Marienkrankenhaus Hamburg, Hamburg, Germany; 2 Department of Obstetrics and Gynecology, Heinrich-Heine-University Düsseldorf, Moorenstr. 5, 40225 Düsseldorf, Germany; 3 Department of Gynecology and Obstetrics, University Hospital Ulm, Ulm, Germany; 4 Department of Obstetrics and Gynecology, University Hospital Essen, University of Duisburg-Essen, Essen, Germany; 5 Department of Gynecology and Obstetrics, University Erlangen, Erlangen, Germany; 6 Department of Gynecology, University Medical Center Hamburg-Eppendorf, Hamburg, Germany; 7 Department of Tumour Biology, University Medical Center Hamburg-Eppendorf, Hamburg, Germany; 8 Department of Gynecology and Obstetrics, Saarland University Hospital, Homburg/Saar, Germany

**Keywords:** Breast cancer, RAS p21, RAS, Circulating tumor cell, Survival, Biomarker

## Abstract

**Background:**

An important component of the RAS signalling pathway, the RAS p21 oncogene, is frequently hyperactivated in breast cancer. Its expression in tumor tissue has been linked to poor clinical outcome. This study was designed to evaluate the clinical relevance of RAS p21 levels in peripheral blood in a large cohort of metastatic breast cancer patients.

**Methods:**

Two hundred fifty-one patients with metastatic breast cancer were enrolled in this prospective, multicentre, open-label, non-randomized study. Blood samples were collected before start of first-line or later-line treatment. RAS p21 was determined using a sandwich-type ELISA immunoassay. For the determination of the cutoff, blood samples from age-matched healthy controls were analyzed. A value above 452 pg/ml was regarded as elevated (mean + 2 x SD). In the univariate survival analysis, two other cutoffs were considered as well (50th and 75th percentile of patients, i.e. 229 pg/ml and 320 pg/ml). Circulating tumor cells (CTCs) were detected using the CellSearch system.

**Results:**

29 of 251 (12%) patients had RAS p21 levels above the cut-off level of 452 pg/ml. Clinical-pathological parameters, such as hormone receptor and HER2 status, line of therapy and CTC status, did not correlate with RAS p21 levels. Elevated RAS p21 was significantly associated with shorter progression-free and overall survival in the univariate analysis (median PFS: 3.9 months [95%-CI: 1.8–6.0] for patients with elevated RAS p21 levels versus 8.5 months [95%-CI: 7.4–9.5] with non-elevated levels [*p* = 0.01]; median OS: 7.1 months [95%-CI: 0.3–14.2] versus not reached [*p* = 0.002], respectively). When RAS p21 cutoffs other than 452 pg/ml were considered, elevated RAS p21 was significantly associated with OS but not with PFS. Classical clinical-pathological factors were included into a multivariate Cox regression analysis. In addition, factors previously shown to influence survival in a univariate analysis, such as serum HER2, CAIX and TIMP1, were included as well. In the multivariate analysis, RAS p21, presence of ≥5 CTCs per 7.5 ml blood, higher grading and higher line of therapy remained independent predictors of shorter OS.

**Conclusions:**

Metastatic breast cancer patients with elevated levels of circulating RAS p21 have significantly worse clinical outcome. Hypothetically, these patients might benefit from therapeutic strategies targeting RAS pathway.

**Trial registration:**

Current Controlled Trials ISRCTN59722891 (DETECT); trial registration date: April, 17th 2010; the trial was registered retrospectively.

## Background

In the past decades, the role of oncogenes and tumor-suppressor genes in the development and progression of breast cancer has attracted considerable research interest. One of the oncogenes influencing cellular signal transduction and controlling such processes as cell differentiation, migration, adhesion and apoptosis, is the RAS family, including three proto-oncogenes: H-Ras, K-Ras, and N-Ras. Oncogenic RAS mutations occur in nearly one-third of all tumor types, making the RAS pathway one of the most commonly deregulated pathways in human carcinoma [1]. Beyond mutations in the RAS genes, upstream regulators and downstream effectors are also able to influence and interact with the pathway.

While RAS mutations are very common in such tumor entities as pancreatic adenocarcinoma, colon cancer, melanoma and leukemia, their prevalence in breast cancer is low [2–5]. According to the current evidence, RAS is mutated in only 1–3% of all malignant breast lesions [6, 7]. Despite gene mutations being a relatively rare event, the RAS pathway is hyperactivated in more than 50% of breast tumors [8–10] and down-regulation of tumor suppressors, such as RasGAP, has been linked to a more aggressive behavior of the disease [11, 12].

Within the RAS gene family, the biological significance of a group of closely related proteins with a special affinity for guanine nucleotides has been the subject of intense research activity. These membrane-associated, nucleotide-binding proteins are referred to as RAS p21, based on their molecular weight of 21,000 Da, and become oncogenic when mutated or overexpressed. RAS p21 levels may be measured in the tumor tissue and, when released from the tumor, in peripheral blood [13, 14]. Data on the prognostic relevance of RAS p21 in breast cancer have yielded conflicting results, with some studies reporting an association between elevated RAS p21 in tumor tissue and poor clinical outcome, while others found no such link [15–17]. With regard to circulating RAS p21, several studies reported significantly higher RAS p21 levels in blood samples of cancer patients than in healthy controls, one of them specifically focusing on breast cancer [14, 18]. However, the impact on survival in breast cancer patients has not been investigated so far.

The aim of the present study was (1) to evaluate the clinical relevance of RAS p21 levels and (2) to compare it to the established biomarker, the circulating tumor cells (CTCs), in patients with metastatic breast cancer.

## Methods

Two hundred fifty-one metastatic breast cancer patients from nine German Breast Cancer Centres were enrolled in this prospective, multicentre, open-label, non-randomized study. Blood was drawn before the start of a new line of therapy. Further inclusion criteria were: age 18 years and older, and first diagnosis of metastatic disease or disease progression before start of a new treatment line. Patients with a second primary malignancy (except in situ carcinoma of the cervix or adequately treated cutaneous basal cell carcinoma) were excluded. Blood samples were collected before start of a new line of therapy chosen according to national and institutional standards. Response to therapy was evaluated by computed tomography every 12 weeks. Informed consent was obtained from all individual participants included in the study.

### Quantitative analysis of RAS p21

RAS p21 was quantified by a commercially available ELISA (Oncogene Science, formerly Siemens Medical Solutions Diagnostics, now Nuclea Biotechnologies Inc., MA, USA) [19]. This sandwich-type immunoassay uses a mouse monoclonal capture antibody and a biotinylated mouse monoclonal antibody as detector. The capture antibody has been immobilized on the interior surface of the microtiter plate wells. After incubating the specimen in the wells to allow binding of the antigen by the capture antibody, the immobilized antigen is exposed to the biotinylated detector antibody. A streptavidin-HRP conjugate is then added. Addition of substrate to the wells allows the catalysis of a chromogen into a colored product, the intensity of which is proportional to the amount of RAS p21 that is bound to the plate. The absorbance of the colored product in the standards and sample wells can be measured using a microtiter plate reader. Currently, of the two monoclonal antibodies used in our assay, only the detector antibody is commercially available (currently as RAS10, catalog # 05–516, MilliporeSigma, Merck, Germany).

For the determination of the cutoff, blood samples from 48 age-matched healthy controls were analyzed (Table 1). The RAS p21 concentration was estimated from the standard curve. A value above 452 pg/ml was regarded as elevated (mean + 2 x SD) [20]. In the univariate survival analysis, two other cutoffs were considered as well (50th and 75th percentile of patients, i.e. 229 pg/ml and 320 pg/ml, respectively). Each sample, standard and control were analyzed in duplicate. Inter-assay and intra-assay coefficients of variation for serum assays were less than 10%.

**Table 1:** Evaluation of RAS p21 in blood samples of healthy controls

Number of controls	48
Mean	200,88 pg/ml
Median	161,00 pg/ml
Range	58–646 pg/ml
Standard deviation	125,56 pg/ml
Mean + 2 SD	452 pg/ml

### Detection of other biomarkers

Circulating tumor cell (CTCs) were detected using the CellSearch™ system (Veridex LLC, NJ, USA). Briefly, 7.5 ml peripheral blood were collected into CellSave Tubes and processed according to manufacturer’s instructions. The assay consists of an immunomagnetic enrichment step employing immunomagnetic beads coated with anti-epithelial cell adhesion molecule (EpCAM) antibody, followed by staining with several antibodies. A circulating tumor cell is defined as a CD45-negative cytokeratin-positive cell with a DAPI-stained nucleus. In the current study, CTC-positive patients were defined as those with at least five tumor cells per 7.5 ml blood with a demonstrated prognostic relevance [21]. Serum HER2 was determined using a commercially available enzyme-linked immunosorbent assay (ELISA) (Martell Diagnostic Laboratories, Roseville, MN, USA; formerly Wilex Inc., Cambridge, MA, USA), as described previously [22]. This test is based on the quantitative measurement of the extracellular domain of the HER2 protein and uses one mouse monoclonal antibody to capture the extracellular domain and another one to detect and quantify it. The assay has been cleared by the Food & Drug Administration (FDA) with the recommended cut-off of 15 ng/ml. Serum TIMP1 and CAIX were quantified by commercially available ELISA (Oncogene Science, formerly Siemens Medical Solutions Diagnostics, now Nuclea Biotechnologies Inc., MA, USA) [23].

### Statistical analysis

Chi-squared test and Fisher’s exact test were used to evaluate the relationship between RAS p21 detection and clinical-pathological factors. In the survival analysis, following primary end points were considered: 1) death and 2) progression. Survival intervals were measured from the time of blood sampling to the time of death or of the first clinical, histological or radiographic diagnosis of progression. We constructed Kaplan–Meier curves and used the log-rank test to assess the univariate significance of the parameters. Cox regression analysis was used for multivariate analysis. All reported *p*-values are two-sided. Statistical analysis was performed by SPSS, version 18 (SPSS Inc., Chicago, IL, USA). The analysis was performed according to the REporting recommendations for tumor MARKer prognostic studies (REMARK) criteria on reporting of biomarkers [24] (Table 2). The primary question was the prognostic impact of RAS p21 in the entire patient cohort.

**Table 2:** REMARK checklist

Item to be reported		Page no.
INTRODUCTION		
1	State the marker examined, the study objectives, and any pre-specified hypotheses.	5
MATERIALS AND METHODS		
*Patients*		
2	Describe the characteristics (e.g., disease stage or co-morbidities) of the study patients, including their source and inclusion and exclusion criteria.	6
3	Describe treatments received and how chosen (e.g., randomized or rule-based).	6
*Specimen characteristics*		
4	Describe type of biological material used (including control samples) and methods of preservation and storage.	6
*Assay methods*		
5	Specify the assay method used and provide (or reference) a detailed protocol, including specific reagents or kits used, quality control procedures, reproducibility assessments, quantitation methods, and scoring and reporting protocols. Specify whether and how assays were performed blinded to the study endpoint.	6–7
*Study design*		
6	State the method of case selection, including whether prospective or retrospective and whether stratification or matching (e.g., by stage of disease or age) was used. Specify the time period from which cases were taken, the end of the follow-up period, and the median follow-up time.	6
7	Precisely define all clinical endpoints examined.	7
8	List all candidate variables initially examined or considered for inclusion in models.	9
9	Give rationale for sample size; if the study was designed to detect a specified effect size, give the target power and effect size.	6
*Statistical analysis methods*		
10	Specify all statistical methods, including details of any variable selection procedures and other model-building issues, how model assumptions were verified, and how missing data were handled.	7
11	Clarify how marker values were handled in the analyses; if relevant, describe methods used for cutpoint determination.	6 Tab. 1
RESULTS		
*Data*		
12	Describe the flow of patients through the study, including the number of patients included in each stage of the analysis (a diagram may be helpful) and reasons for dropout. Specifically, both overall and for each subgroup extensively examined report the numbers of patients and the number of events.	8 Fig. 1
13	Report distributions of basic demographic characteristics (at least age and sex), standard (disease-specific) prognostic variables, and tumor marker, including numbers of missing values.	8
*Analysis and presentation*		
14	Show the relation of the marker to standard prognostic variables.	8–9 Tab. 2
15	Present univariable analyses showing the relation between the marker and outcome, with the estimated effect (e.g., hazard ratio and survival probability). Preferably provide similar analyses for all other variables being analyzed. For the effect of a tumor marker on a time-to-event outcome, a Kaplan-Meier plot is recommended.	8 Fig. 2a,b
16	For key multivariable analyses, report estimated effects (e.g., hazard ratio) with confidence intervals for the marker and, at least for the final model, all other variables in the model.	9 Tab. 3
17	Among reported results, provide estimated effects with confidence intervals from an analysis in which the marker and standard prognostic variables are included, regardless of their statistical significance.	8–9
18	If done, report results of further investigations, such as checking assumptions, sensitivity analyses, and internal validation.	
DISCUSSION		
19	Interpret the results in the context of the pre-specified hypotheses and other relevant studies; include a discussion of limitations of the study.	10–13
20	Discuss implications for future research and clinical value.	10–13

## Results

### Patients’ characteristics

**Fig. 1:**
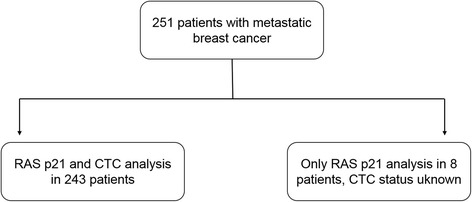
Patient distribution diagram according to the REMARK criteria

Two hundred fifty-one patients diagnosed with metastatic breast cancer were included into the analysis. Clinical-pathological data are summarized in Table 3. The median age of patients was 60 years. 70% of patients had ER-positive tumors. HER2 was overexpressed by the primary tumor and/or metastasis in 35% of patients. Visceral metastases were present in 39% of patients, bone metastases in 14%; 47% of patients had both visceral and bone involvement. 79% of patients received chemotherapy, 21% endocine therapy and 22% trastuzumab during the trial (Table 4). At least five CTCs per 7.5 ml of peripheral blood were detected in 122 of 243 evaluable patients (50%). The distribution of patients is summarized in a REMARK diagram (Fig. 1).

**Table 3:** Patients’ characteristics

	RAS p21		
	Total	RAS p21 elevated *n* (%)	*p*-value
Overall	251	29 (12%)	
ER status			0.611
Negative	76	10 (13%)	
Positive	174	19 (11%)	
PR status			0.358
Negative	101	14 (14%)	
Positive	149	15 (10%)	
HER2 status			0.873
Negative^a^	143	18 (13%)	
Positive^b^	76	9 (12%)	
Tumor subtype			0.728
Triple-negative	37	6 (16%)	
HR-positive HER2-negative	106	12 (11%)	
HER2-positive	76	9 (12%)	
Metastatic site			0.482
Visceral	98	13 (13%)	
Bone	35	2 (6%)	
Both	118	14 (12%)	
Extent of metastatic disease			0.768
One site	84	9 (11%)	
Multiple sites	167	20 (12%)	
Therapeutic setting			0.249
1st-line	98	8 (8%)	
2nd-line	66	11 (17%)	
3rd-line or more	86	10 (12%)	
Grading			0.604
G1	5	1 (20%)	
G2	129	13 (10%)	
G3	103	14 (14%)	
Circulating tumor cells			0.101
< 5 CTCs / 7.5 ml	122	17 (14%)	
≥ 5 CTCs / 7.5 ml	121	9 (7%)	

Abbreviations: *HR* hormone receptor

^a^IHC score: 0 /+ 1 or FISH negative

^b^IHC score: + 3 or FISH positive

**Table 4:** Detailed analysis of therapy administered during the trial

	Chemotherapy	Endocrine therapy	Trastuzumab
ER status			
Negative	63 / 76 (83%)	3 / 76 (4%)	20 / 76 (26%)
Positive	135 / 174 (78%)	50 / 174 (29%)	35 / 174 (20%)
PR status			
Negative	81 / 101 (80%)	13 / 101 (13%)	27 / 101 (27%)
Positive	117 / 149 (79%)	40 / 149 (27%)	28 / 149 (19%)
HER2 status			
Negative	112 / 143 (78%)	34 / 143 (24%)	3 / 144 (2%)
Positive	66 / 76 (87%)	7 / 76 (9%)	44 / 77 (57%)
Circulating tumor cells			
≥ 5 CTCs / 7.5 ml	100 / 122 (82%)	25 / 122 (21%)	19 / 122 (16%)
< 5 CTCs / 7.5 ml	94 / 123 (76%)	26 / 123 (21%)	34 / 123 (28%)
RAS p21			
Elevated	25 / 29 (86%)	2 / 29 (7%)	9 / 29 (31%)
Non elevated	174 / 222 (78%)	51 / 222 (23%)	46 / 222 (21%)

### RAS p21 detection

29 of 251 (12%) patients had RAS p21 levels above the cut-off level of 452 pg/ml. Clinical-pathological parameters, such as hormone receptor and HER2 status, line of therapy, extent of disease and grading, did not correlate with RAS p21 levels (Table 3). The CTC status was not associated with RAS p21 (*p* = 0.101).

### Univariate survival analysis

**Fig. 2:**
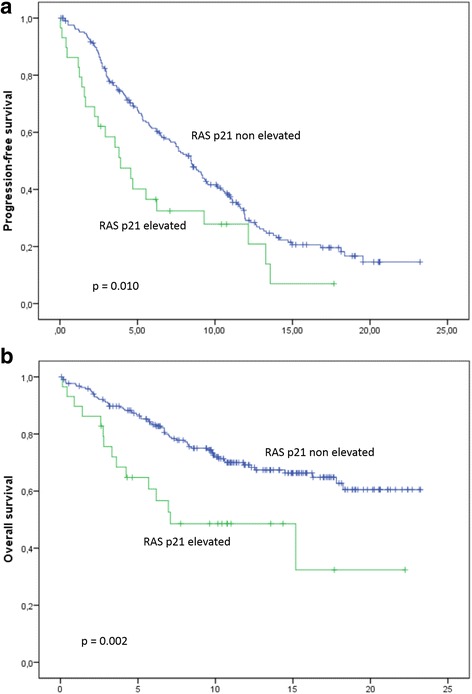
Correlation between RAS p21 levels and progression-free (**a**) and overall survival (**b**) [months]

During a median follow up of 19 months, 85 patients died and 183 were diagnosed with progressive disease. Median progression-free survival (PFS) was 3.9 months (95%-CI: 1.8–6.0 months) for patients with elevated RAS p21 levels versus 8.5 months (7.4–9.5 months) with non-elevated levels (*p* = 0.01) (Fig. 2a). Median overall survival (OS) was 7.1 months (0.3–14.2 months) in patients with elevated RAS p21; median OS in patients with non-elevated RAS p21 has not been reached (*p* = 0.002) (Fig. 2b). As reported previously, the CTC status was significantly associated with shorter PFS (*p* = 0.001) and OS (*p* <  0.001). Patients with elevated RAS p21 and ≥ 5 CTCs had median PFS of 4.6 months (2.4–6.8) and median OS of 7.0 months (5.3–8.6), whereas those with non-elevated RASp21 and < 5 CTCs had PFS of 10.4 (8.7–12.2) months; median OS was not reached (*p* <  0.001 for PFS and OS). When RAS p21 cutoffs other than 452 pg/ml (mean + 2SD) were considered, elevated RAS p21 was significantly associated with OS (*p* = 0.019 in case of 229 pg/ml; *p* = 0.003 in case of 320 pg/ml), but not with PFS (*p* = 0.623 in case of 229 pg/ml; *p* = 0.153 in case of 320 pg/ml). When stratified by tumor subtype, RAS p21 correlated significantly with OS and PFS in HER2-positive patients (*p* < 0.001 for OS and *p* = 0.001 for PFS, respectively). In patients with hormone receptor positive HER2-negative tumors, we found borderline significance for OS (*p* = 0.054) and no significance for PFS (*p* = 0.517). In the subgroup of patients with triple-negative breast cancer, no correlation was found between survival and RAS p21 (*p* = 0.811 for OS and *p* = 0.679 for PFS). When stratified by therapy received during the study, RAS p21 was significantly associated with OS in ER-positive patients receiving endocrine therapy (*p* = 0.004) and in HER2-positive patients receiving trastuzumab (*p* = 0.009). The group of ER-positive patients not receiving endocrine therapy was too small to perform statistical analysis and none of the HER2-positive patients not receiving trastuzumab had elevated RAS p21 levels. With regard to PFS, the impact of RAS p21 remained significant in the group of ER-positive patients receiving endocrine treatment (*p* = 0.009) and HER2-positive patients receiving trastuzumab (*p* < 0.001).

### Multivariate survival analysis

The variables for the multivariate Cox regression analysis were identified via backward selection. Besides classical prognostic factors, biomarkers previously shown to influence survival in a univariate analysis, such as serum HER2, CAIX and TIMP1, were included in the multivariate model (*p*-values in the univariate survival analyses were as follows: serum HER2: 0.001 for OS and 0.077 for PFS; CAIX: < 0.001 for both OS and PFS; TIMP1: < 0.001 for both OS and PFS). After adjusting for these variables, RAS p21, presence of ≥5 CTCs, higher grading and higher line of therapy remained independent predictors of shorter OS (Table 5). Negative ER status, higher line of therapy and elevated CTC counts were independent predictors of shorter PFS in the multivariate analysis.

**Table 5:** Multivariate analysis of overall survival

	*p*-value	Hazard Ratio	95%-Confidence Interval
RAS p21 Elevated vs. non elevated	0.003	2.927	1.43–5.99
CTC counts ≥5 vs. < 5 CTCs / 7.5 ml blood	< 0.001	3.775	2.01–7.10
Therapy line > 1st line vs. 1st line	0.002	2.817	1.48–5.37
Grading G3 vs. G1/2	0.026	1.380	1.04–1.83
Serum HER2 Elevated vs. non elevated	0.156	1.564	0.84–2.90
Menopausal status Post- vs. Premenopausal	0.298	0.732	0.41–1.32
ER status Positive vs. Negative	0.067	0.478	0.22–1.05
PR status Positive vs. Negative	0.486	1.297	0.62–2.70
HER2 status Positive vs. Negative	0.121	0.623	0.34–1.13
Number of metastatic sites Multiple vs. Single site	0.521	1.262	0.62–2.57
Metastatic spread Visceral (+/−) vs. bone only	0.567	1.566	0.33–7.54
CAIX Elevated vs. non elevated	0.300	1.381	0.75–2.54
TIMP1 Elevated vs. non elevated	0.066	1.741	0.96–3.15

## Discussion

To our knowledge, this is the first study to address the clinical relevance of circulating RAS p21 in a large group of metastatic breast cancer patients. Using a cutoff derived from a cohort of healthy controls, elevated RAS p21 levels were detected in 12% of patients and independently predicted shorter overall survival.

The RAS proteins were some of the first proteins identified able to regulate cellular growth [1]. Human cancers frequently express activating mutations in the RAS genes, which have been linked to several malignant characteristics of tumor cells, such as deregulated proliferation, invasiveness and neo-angiogenesis, making the RAS pathway an attractive target for new oncological strategies [25]. Interestingly, breast cancer cells are able to activate RAS signalling pathways by a variety of mechanisms, although they generally lack RAS mutations [6]. Several growth factor receptors, such as erbB-1/2, IGF-1 and ERα, have been described to interfere with the pathway and stimulate RAS proteins [1, 26]. The loss of tumor suppressors of the RAS pathway has been recently shown to be associated with a more aggressive phenotype [11]. Among the proteins encoded by members of the RAS gene family, a group of closely related proto-oncogenes with a molecular weight of 21,000 Da is referred to as RAS p21. Localized in the cytoplasmic surface of the cell membrane, these molecules serve as transducers for signals involved in cell proliferation.

### Histopathological detection of tissue RAS p21

So far, most of the available data on RAS p21 in human carcinoma are based on studies examining its detection in tumor tissue. RAS p21 in the context of breast cancer was first described in 1984, with Hand et al. demonstrating that 90% of invasive ductal carcinomas show positive staining for RAS p21 [27], followed by reports on the expression in primary and metastatic breast cancer [28, 29]. The majority of studies assessed RAS p21 using a monoclonal antibody Y13–259 and reported a stronger staining intensity in malignant breast lesions than in the adjacent tissue or in benign and inflammatory lesions, with the exception of proliferating fibroadenoma and complex cystic disease, which generally showed at least a weak staining [30, 31]. In contrast, one group found positive staining in normal breast tissue and benign non-proliferative tumors as well; however, the intensity of staining was not evaluated in a systematic manner [32]. Going et al. proposed a scoring system based on the staining extent and intensity and demonstrated the highest staining scores in malignant breast lesions, followed by atypical ductal hyperplasia, ductal hyperplasia without atypias and finally normal breast tissue [33]. The evidence on the correlation of RAS p21 tissue expression with conventional prognostic parameters is inconclusive; some studies reported an association between higher RAS p21 expression and positive lymph node status [34], while others found stronger RAS p21 staining in patients younger than 50 years and with estrogen receptor negative tumors [33, 35].

### The prognostic relevance of tissue RAS p21

Ohuchi et al. reported on a long-term follow up of a small group of women with hyperplastic breast lesions with or without atypia [28]. Five of 18 women were diagnosed with ipsilateral breast cancer during 15 years of follow up. RAS p21 expression in women who developed cancer was significantly higher than in those who did not. In one of the older studies, Dati et al. examined tumors from 132 primary breast cancer patients and showed that high RAS p21 levels predicted worse clinical outcome [36]; elevated RAS p21 correlated with overexpression of c-erbB-2 encoded p185 protein, indicating a possible cross-talk between the erbB-2 and RAS signalling cascade. In contrast, Archer et al. analysed the expression of various oncogenes in tissue samples from 92 patients with advanced or metastatic breast cancer and reported that RAS p21 positivity did not correlate with PFS or OS [15]. However, the staining pattern in this study was not quantitatively assessed and patients were stratified in two categories (RAS p21 positive and negative), the majority (72%) belonging to the first group. Further, the trial was designed to assess the response to endocrine therapy received by all patients, although 40% of the tumors were ER-negative and 59% were PR-negative.

### Detection of RAS p21 in peripheral blood

Since overexpressed proteins are frequently shed by malignant cells into the blood, numerous studies evaluated the possibility of measuring circulating RAS p21 in plasma samples of cancer patients. However, only few addressed this issue specifically in breast cancer. Weissfeld et al. analysed plasma samples from 80 patients with various solid cancers and 286 healthy subjects and found significantly higher levels of circulating RAS p21 in cancer patients; no differences in RAS p21 expression between tumor sites were observed [18]. In haematological malignancies, such as acute leukemia, RAS p21 can be detected and quantified in bone marrow samples; in myelodysplastic syndrome, elevated levels have been linked to disease progression [37, 38]. It has been speculated whether serum detection of RAS p21 might contribute to early detection of lung cancer in persons particularly at risk [39].

The only study focussing specifically on RAS p21 expression in the blood of breast cancer patients was conducted using Western blot analysis and a mouse monoclonal antibody 142-24E05 which detects both normal and mutant RAS protein [14]. RAS p21 was measured in samples from 34 newly diagnosed early breast cancer patients, 26 women with benign breast disease and 34 healthy women. RAS p21 was detected in 53% of cancer cases, 27% of women with benign lesions and 26% of the healthy controls. After adjusting for possible confounders, such as age, parity, familial risk and hormonal factors, RAS p21 remained an independent predictor of cancer status. Interestingly, four of five patients with ductal carcinoma in situ had detectable RAS p21, indicating that RAS pathway becomes activated at earliest stages of the disease.

In the present study, RAS p21 levels in the peripheral blood were measured using a sandwich-type ELISA. Controlling for established clinical-pathological parameters, elevated RAS p21 independently predicted shorter overall survival. To place RAS p21 detection in context of more extensively studied blood-based biomarkers, CTC status, serum HER2, CAIX and TIMP1 were included in the analysis as well [22, 23]. RAS p21 and CTC status were the only biomarkers associated with shorter OS in the multivariate analysis. Interestingly, detection of ≥5 CTCs per 7.5 ml blood was not associated with elevated RAS p21, suggesting that the presence of RAS p21 is not a mere epiphenomenon of CTCs but has a clinical relevance of its own. While oncogene products are frequently shed into blood circulation, it remains unclear whether high levels of RAS p21 in patients with poor prognosis are released by metastatic lesions or by the CTCs. Since overexpression of RAS p21 in the tumor tissue has been linked to worse survival, it might be hypothesized that the origin of circulating RAS p21 is the metastasis; however, no study so far examined the expression of RAS p21 in both tumor tissue and blood in the same subset of breast cancer patients and evidence from other entities is scarce. In colon carcinoma, a statistically significant correlation for RAS p21 overexpression was reported in matched tissue and plasma samples [40]. In a small study on liver angiosarcoma, 80% of tumors had a specific RAS mutation and were found to express the corresponding mutant p21 protein in their tumor tissue and serum [41].

Whether the RAS cascade is activated/deregulated more frequently in some subtypes of breast cancer than in others, remains unclear. Fribbens et al. examined plasma samples from a homogenous collective of 113 hormone receptor positive patients who progressed after at least six months of aromatase inhibitor therapy [42]. In 21% of samples sub-clonal KRAS mutations in circulating tumor DNA were detected, suggesting a potential role for selected mutations in resistance to endocrine therapy. Interestingly, the clinical outcome was not associated with the presence of KRAS mutations. While the mutation status has not been analysed in our study, we could show the highest clinical relevance of RAS p21 in HER2-positive patients, followed by the hormone receptor positive HER2-negative group. In the triple-negative subgroup, survival of patients with elevated and non-elevated RAS p21 was similar, suggesting that the biological significance of RAS signalling cascade is highest in cancers with non-triple negative phenotype.

### Clinical relevance of RAS p21 detection

The correlation between elevated RAS p21 and poor prognosis makes the RAS pathway an attractive candidate for targeted therapies [1]. The precise molecule targeting RAS p21 is unknown; however, potential approaches that have been discussed in this context are the inhibition of RAS p21 expression through antisense oligonucleotides or ribozymes and the inhibition of farnesylation. The latter strategy has initially gained considerable interest, as farnesylation is involved in the posttranslational modification of RAS and is necessary to attach RAS proteins to the cell membrane [43]. Several inhibitors of farnesyltransferase, the crucial enzyme of this process, have been developed but have so far not proven effective in clinical trials.

**Fig. 3:**
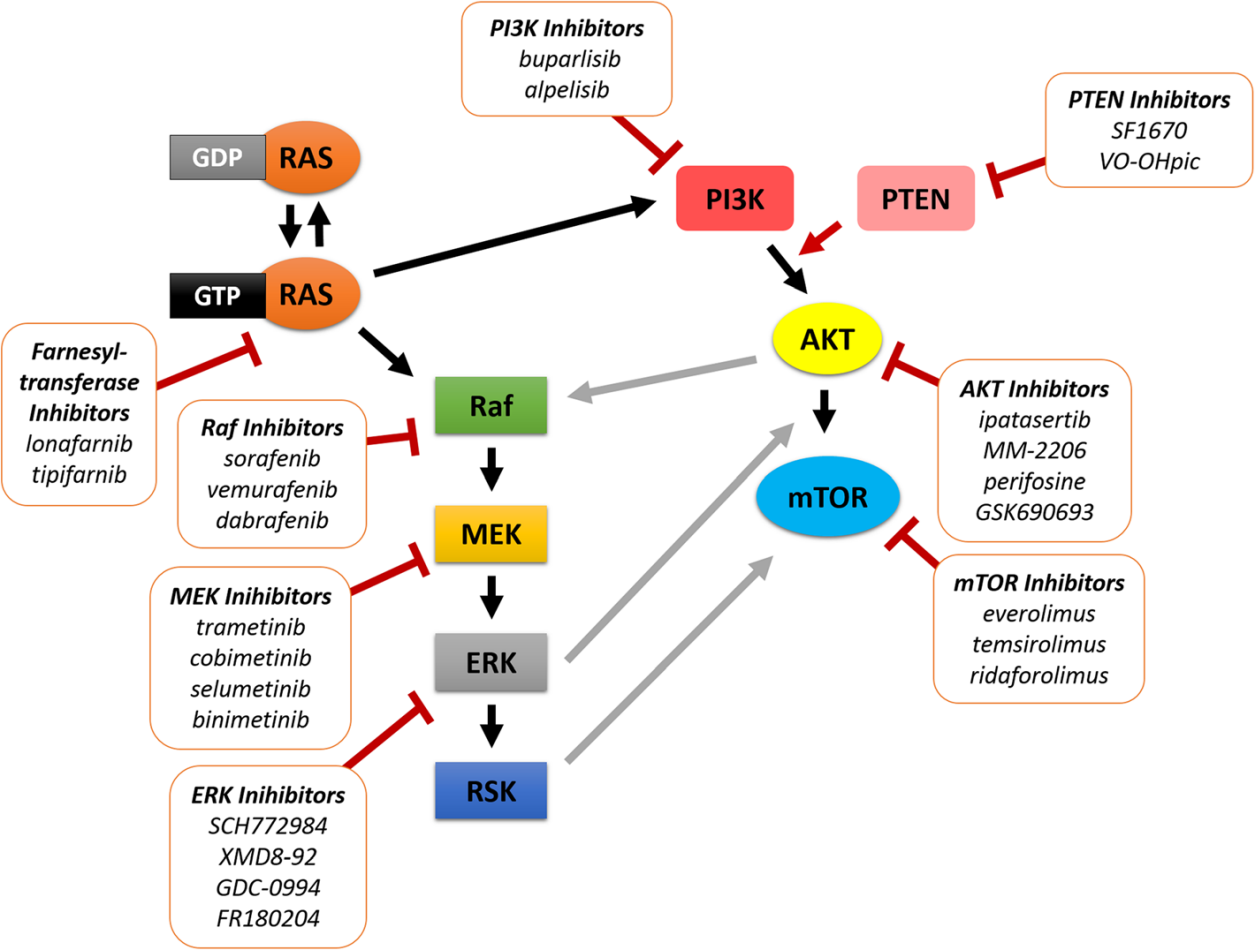
Interactions between main components of the RAS effector pathways and agents targeting specific steps of the signalling cascade. Abbreviations: GTP – guanosine triphosphate; GDP – guanosine diphosphate; ERK – extracellular signal–regulated kinases; MEK – mitogen-activated protein kinase; RSK – ribosomal protein S6 kinase; PI3K – phosphatidylinositide 3-kinase; PTEN – phosphatase and tensin homolog; mTOR – mechanistic target of rapamycin

Other potential strategies aim at targeting effector pathways downstream of RAS (Fig. 3). The Raf-MEK-ERK signal transduction cascade was one of the first RAS-activated pathways identified and plays an important role as a target for Raf and MEK kinase inhibitors [44]. Sorafenib, a Raf kinase inhibitor, is effective in renal cell and hepatocellular carcinoma, but failed to show clinical benefit in breast cancer [45]. Another effector pathway interfering with the RAS family is the PI3K/AKT/mTOR pathway [46]. Since the efficacy of inhibitors of a single pathway is often reduced by the negative feedback loops activating the second pathway, dual blockade of both pathways may be necessary to inhibit tumor growth [47].

## Conclusions

Detection of elevated RAS p21 levels in the peripheral blood of metastatic breast cancer patients provides important prognostic information. Hypothetically, these patients might benefit from agents targeting components of the RAS signaling pathway. Whether changes in RAS p21 status might potentially improve therapy monitoring, remains to be clarified in future trials.

## Abbreviations

CTC: Circulating tumor cell
ELISA: Enzyme-linked immunosorbent assay
EpCAM: Epithelial cell adhesion molecule
FDA: Food & Drug Administration
HER2: Human epidermal growth factor receptor 2
OS: Overall survival
PFS: Progression-free survival
REMARK: REporting recommendations for tumor MARKer prognostic studies

## References

[1] Downward J (2003). Targeting RAS signalling pathways in cancer therapy. Nat Rev Cancer.

[2] Zhang J, Ding L, Holmfeldt L, Wu G, Heatley SL, Payne-Turner D, Easton J, Chen X, Wang J, Rusch M (2012). The genetic basis of early T-cell precursor acute lymphoblastic leukaemia. Nature.

[3] Cancer Genome Atlas N (2012). Comprehensive molecular characterization of human colon and rectal cancer. Nature.

[4] Biankin AV, Waddell N, Kassahn KS, Gingras MC, Muthuswamy LB, Johns AL, Miller DK, Wilson PJ, Patch AM, Wu J (2012). Pancreatic cancer genomes reveal aberrations in axon guidance pathway genes. Nature.

[5] Hodis E, Watson IR, Kryukov GV, Arold ST, Imielinski M, Theurillat JP, Nickerson E, Auclair D, Li L, Place C (2012). A landscape of driver mutations in melanoma. Cell.

[6] Gao J, Aksoy BA, Dogrusoz U, Dresdner G, Gross B, Sumer SO, Sun Y, Jacobsen A, Sinha R, Larsson E (2013). Integrative analysis of complex cancer genomics and clinical profiles using the cBioPortal. Sci Signal.

[7] Bamford S, Dawson E, Forbes S, Clements J, Pettett R, Dogan A, Flanagan A, Teague J, Futreal PA, Stratton MR (2004). The COSMIC (catalogue of somatic mutations in Cancer) database and website. Br J Cancer.

[8] Sivaraman VS, Wang H, Nuovo GJ, Malbon CC (1997). Hyperexpression of mitogen-activated protein kinase in human breast cancer. J Clin Invest.

[9] von Lintig FC, Dreilinger AD, Varki NM, Wallace AM, Casteel DE, Boss GR (2000). Ras activation in human breast cancer. Breast Cancer Res Treat.

[10] Mueller H, Flury N, Eppenberger-Castori S, Kueng W, David F, Eppenberger U (2000). Potential prognostic value of mitogen-activated protein kinase activity for disease-free survival of primary breast cancer patients. Int J Cancer.

[11] Olsen SN, Wronski A, Castano Z, Dake B, Malone C, De Raedt T, Enos M, DeRose YS, Zhou W, Guerra S (2017). Loss of RasGAP tumor suppressors underlies the aggressive nature of luminal B breast cancers. Cancer Discov.

[12] Barras D, Lorusso G, Lhermitte B, Viertl D, Ruegg C, Widmann C (2014). Fragment N2, a caspase-3-generated RasGAP fragment, inhibits breast cancer metastatic progression. Int J Cancer.

[13] Shih TY, Hattori S, Clanton DJ, Ulsh LS, Chen ZQ, Lautenberger JA, Papas TS (1986). Structure and function of p21 ras proteins. Gene Amplif Anal.

[14] Rundle A, Tang D, Brandt-Rauf P, Zhou J, Kelly A, Schnabel F, Perera FP (2002). Association between the ras p21 oncoprotein in blood samples and breast cancer. Cancer Lett.

[15] Archer SG, Eliopoulos A, Spandidos D, Barnes D, Ellis IO, Blamey RW, Nicholson RI, Robertson JF (1995). Expression of ras p21, p53 and c-erbB-2 in advanced breast cancer and response to first line hormonal therapy. Br J Cancer.

[16] Clair T, Miller WR, Cho-Chung YS (1987). Prognostic significance of the expression of a ras protein with a molecular weight of 21,000 by human breast cancer. Cancer Res.

[17] Watson DM, Elton RA, Jack WJ, Dixon JM, Chetty U, Miller WR (1991). The H-ras oncogene product p21 and prognosis in human breast cancer. Breast Cancer Res Treat.

[18] Weissfeld JL, Larsen RD, Niman HL, Kuller LH (1994). Evaluation of oncogene-related proteins in serum. Cancer Epidemiol Biomark Prev.

[19] Hamer PJ, Trimpe KL, Pullano T, Ng S, LaVecchio JA, Petit DA, DeLellis R, Wolfe H, Carney WP (1990). Production and characterization of anti-RAS p21 monoclonal antibodies. Hybridoma.

[20] Carney WP (2007). Circulating oncoproteins HER2/neu, EGFR and CAIX (MN) as novel cancer biomarkers. Expert Rev Mol Diagn.

[21] Bidard FC, Peeters DJ, Fehm T, Nole F, Gisbert-Criado R, Mavroudis D, Grisanti S, Generali D, Garcia-Saenz JA, Stebbing J (2014). Clinical validity of circulating tumour cells in patients with metastatic breast cancer: a pooled analysis of individual patient data. Lancet Oncol.

[22] Banys-Paluchowski M, Witzel I, Riethdorf S, Rack B, Janni W, Fasching PA, Solomayer EF, Aktas B, Kasimir-Bauer S, Pantel K (2017). Clinical relevance of serum HER2 and circulating tumor cell detection in metastatic breast Cancer patients. Anticancer Res.

[23] Muller V, Riethdorf S, Rack B, Janni W, Fasching PA, Solomayer E, Aktas B, Kasimir-Bauer S, Zeitz J, Pantel K (2011). Prospective evaluation of serum tissue inhibitor of metalloproteinase 1 and carbonic anhydrase IX in correlation to circulating tumor cells in patients with metastatic breast cancer. Breast Cancer Res.

[24] McShane LM, Altman DG, Sauerbrei W, Taube SE, Gion M, Clark GM (2005). REporting recommendations for tumour MARKer prognostic studies (REMARK). Br J Cancer.

[25] Shields JM, Pruitt K, McFall A, Shaub A, Der CJ (2000). Understanding Ras: 'it ain't over 'til it's over. Trends Cell Biol.

[26] Migliaccio A, Piccolo D, Castoria G, Di Domenico M, Bilancio A, Lombardi M, Gong W, Beato M, Auricchio F (1998). Activation of the Src/p21ras/Erk pathway by progesterone receptor via cross-talk with estrogen receptor. EMBO J.

[27] Hand PH, Thor A, Wunderlich D, Muraro R, Caruso A, Schlom J (1984). Monoclonal antibodies of predefined specificity detect activated ras gene expression in human mammary and colon carcinomas. Proc Natl Acad Sci U S A.

[28] Ohuchi N, Thor A, Page DL, Hand PH, Halter SA, Schlom J (1986). Expression of the 21,000 molecular weight ras protein in a spectrum of benign and malignant human mammary tissues. Cancer Res.

[29] Fromowitz FB, Viola MV, Chao S, Oravez S, Mishriki Y, Finkel G, Grimson R, Lundy J (1987). Ras p21 expression in the progression of breast cancer. Hum Pathol.

[30] Agnantis NJ, Apostolikas NA, Zolotas VG, Spandidos DA (1994). Immunohistochemical detection of ras p21 oncoprotein in breast cancer imprints. Acta Cytol.

[31] Agnantis NJ, Petraki C, Markoulatos P, Spandidos DA (1986). Immunohistochemical study of the ras oncogene expression in human breast lesions. Anticancer Res.

[32] Candlish W, Kerr IB, Simpson HW (1986). Immunocytochemical demonstration and significance of p21 ras family oncogene product in benign and malignant breast disease. J Pathol.

[33] Going JJ, Anderson TJ, Wyllie AH (1992). Ras p21 in breast tissue: associations with pathology and cellular localisation. Br J Cancer.

[34] Efremidis AP, Agnantis NJ, Patra F, Papadopoulou C, Spandidos DA (1989). Clinical significance of elevated p21 ras oncogene expression in breast cancer patients. Cancer J.

[35] Spandidos DA, Yiagnisis M, Papadimitriou K, Field JK (1989). Ras, c-myc and c-erbB-2 oncoproteins in human breast cancer. Anticancer Res.

[36] Dati C, Muraca R, Tazartes O, Antoniotti S, Perroteau I, Giai M, Cortese P, Sismondi P, Saglio G, De Bortoli M (1991). C-erbB-2 and ras expression levels in breast cancer are correlated and show a co-operative association with unfavorable clinical outcome. Int J Cancer.

[37] Kalmanti M, Kalmantis T, Vassilaki M, Galanopoulos A, Grenzelias D, Spandidos DA (1992). The expression of the ras p21 oncoprotein in the bone marrow smears of children with acute leukemia. Anticancer Res.

[38] Kalmantis T, Kalmanti M, Vassilaki M, Spandidos DA (1993). Analysis of immunohistochemical results of the ras oncogene product p21 in myelodysplastic syndromes. Anticancer Res.

[39] Brandt-Rauf PW (1991). Oncogene proteins as biomarkers in the molecular epidemiology of occupational carcinogenesis. The example of the ras oncogene-encoded p21 protein. Int Arch Occup Environ Health.

[40] Luo JC, Neugut AI, Garbowski G, Forde KA, Treat M, Smith S, Niman H, Brandt-Rauf PW (1996). Expression of p21ras-related protein in the plasma and tissue of patients with adenomas and carcinomas of the colon. Biomarkers.

[41] De Vivo I, Marion MJ, Smith SJ, Carney WP, Brandt-Rauf PW (1994). Mutant c-Ki-ras p21 protein in chemical carcinogenesis in humans exposed to vinyl chloride. Cancer Causes Control.

[42] Fribbens C, Garcia Murillas I, Beaney M, Hrebien S, O'Leary B, Kilburn L, Howarth K, Epstein M, Green E, Rosenfeld N et al: Tracking evolution of aromatase inhibitor resistance with circulating tumour DNA analysis in metastatic breast cancer. Ann Oncol. 2018;29(1):145-53. 10.1093/annonc/mdx483. 10.1093/annonc/mdx483 PMC6264798 29045530

[43] Rowinsky EK, Windle JJ, Von Hoff DD (1999). Ras protein farnesyltransferase: a strategic target for anticancer therapeutic development. J Clin Oncol.

[44] Gysin S, Salt M, Young A, McCormick F (2011). Therapeutic strategies for targeting ras proteins. Genes Cancer.

[45] Baselga J, Costa F, Gomez H, Hudis CA, Rapoport B, Roche H, Schwartzberg LS, Petrenciuc O, Shan M, Gradishar WJ (2013). A phase 3 tRial comparing capecitabinE in combination with SorafenIb or pLacebo for treatment of locally advanced or metastatIc HER2-negative breast CancEr (the RESILIENCE study): study protocol for a randomized controlled trial. Trials.

[46] Mendoza MC, Er EE, Blenis J (2011). The Ras-ERK and PI3K-mTOR pathways: cross-talk and compensation. Trends Biochem Sci.

[47] Sos ML, Fischer S, Ullrich R, Peifer M, Heuckmann JM, Koker M, Heynck S, Stuckrath I, Weiss J, Fischer F (2009). Identifying genotype-dependent efficacy of single and combined PI3K- and MAPK-pathway inhibition in cancer. Proc Natl Acad Sci U S A.

